# Dietary protein to maximize resistance training: a review and examination of protein spread and change theories

**DOI:** 10.1186/1550-2783-9-42

**Published:** 2012-09-08

**Authors:** John D Bosse, Brian M Dixon

**Affiliations:** 1 USANA Health Sciences, Inc, 3838 West Parkway Boulevard, Salt Lake City, UT, 84120, USA; 2 Division of Nutrition, University of Utah, 250 South 1850 East #214, Salt Lake City, UT, 84112, USA

**Keywords:** Protein, Habitual protein intake, Strength, Muscle hypertrophy, Resistance training

## Abstract

An appreciable volume of human clinical data supports increased dietary protein for greater gains from resistance training, but not all findings are in agreement. We recently proposed “protein spread theory” and “protein change theory” in an effort to explain discrepancies in the response to increased dietary protein in weight management interventions. The present review aimed to extend “protein spread theory” and “protein change theory” to studies examining the effects of protein on resistance training induced muscle and strength gains. Protein spread theory proposed that there must have been a sufficient spread or % difference in g/kg/day protein intake between groups during a protein intervention to see muscle and strength differences. Protein change theory postulated that for the higher protein group, there must be a sufficient change from baseline g/kg/day protein intake to during study g/kg/day protein intake to see muscle and strength benefits. Seventeen studies met inclusion criteria. In studies where a higher protein intervention was deemed successful there was, on average, a 66.1% g/kg/day between group intake spread versus a 10.2% g/kg/day spread in studies where a higher protein diet was no more effective than control. The average change in habitual protein intake in studies showing higher protein to be more effective than control was +59.5% compared to +6.5% when additional protein was no more effective than control. The magnitudes of difference between the mean spreads and changes of the present review are similar to our previous review on these theories in a weight management context. Providing sufficient deviation from habitual intake appears to be an important factor in determining the success of additional protein in enhancing muscle and strength gains from resistance training. An increase in dietary protein favorably effects muscle and strength during resistance training.

## Introduction

As sports nutrition science has evolved in recent decades it has been increasingly common for athletes to use diet and supplementation as tools to enhance their training and performance. With the increase in sports nutrition knowledge has come an array of purported performance enhancing dietary supplements. One of the most common, widely used, and studied classes of supplements is protein powders - traditionally whey, casein, soy, or egg. Studies commonly use supplemental forms of protein rather than whole foods, most likely due to greater shelf stability and the ease of providing participants with protein powder to be consumed in addition to their habitual diet. Compliance is likely easier to monitor as well (counting empty supplement packets), than when participants are entrusted to cook additional food to achieve a target diet. Determining if increases in protein intake are warranted to promote resistance training gains is the focal point of this review. Answering this question involves addressing two key areas: 1) the level of dietary protein intake that has been shown to provide the greatest results in resistance training studies; and 2) whether or not there is a discrepancy between this level of protein intake and habitual protein intakes of participants at baseline in these studies.

Most studies support the utility of increasing protein intake to promote muscular benefits while resistance training [1-10]. While evidence weighs heavily in this direction, as with most areas, data are not entirely conclusive. Recently we proposed protein spread theory and protein change theory as possible explanations for discrepancies within the protein and weight management literature [11]. Whether or not these theories are supported in resistance training studies is unknown. Therefore, the purpose of the present review is to examine our protein spread and change theories in the context of muscle and strength gains from resistance training.

## Methods

Protein spread theory postulated that there must be a sufficient spread or difference in g/kg/day protein intake between groups to see muscle and strength differences. Protein change theory postulates that there must be a sufficient change from baseline g/kg/day protein intake to during study g/kg/day protein intake to see muscle and strength benefits. “Muscular benefits” referred to herein are benefits to the following that were greater than control: lean mass gain, lean mass preservation, strength gain, muscle cross-sectional area gain, and fat loss.

**Figure 1:**
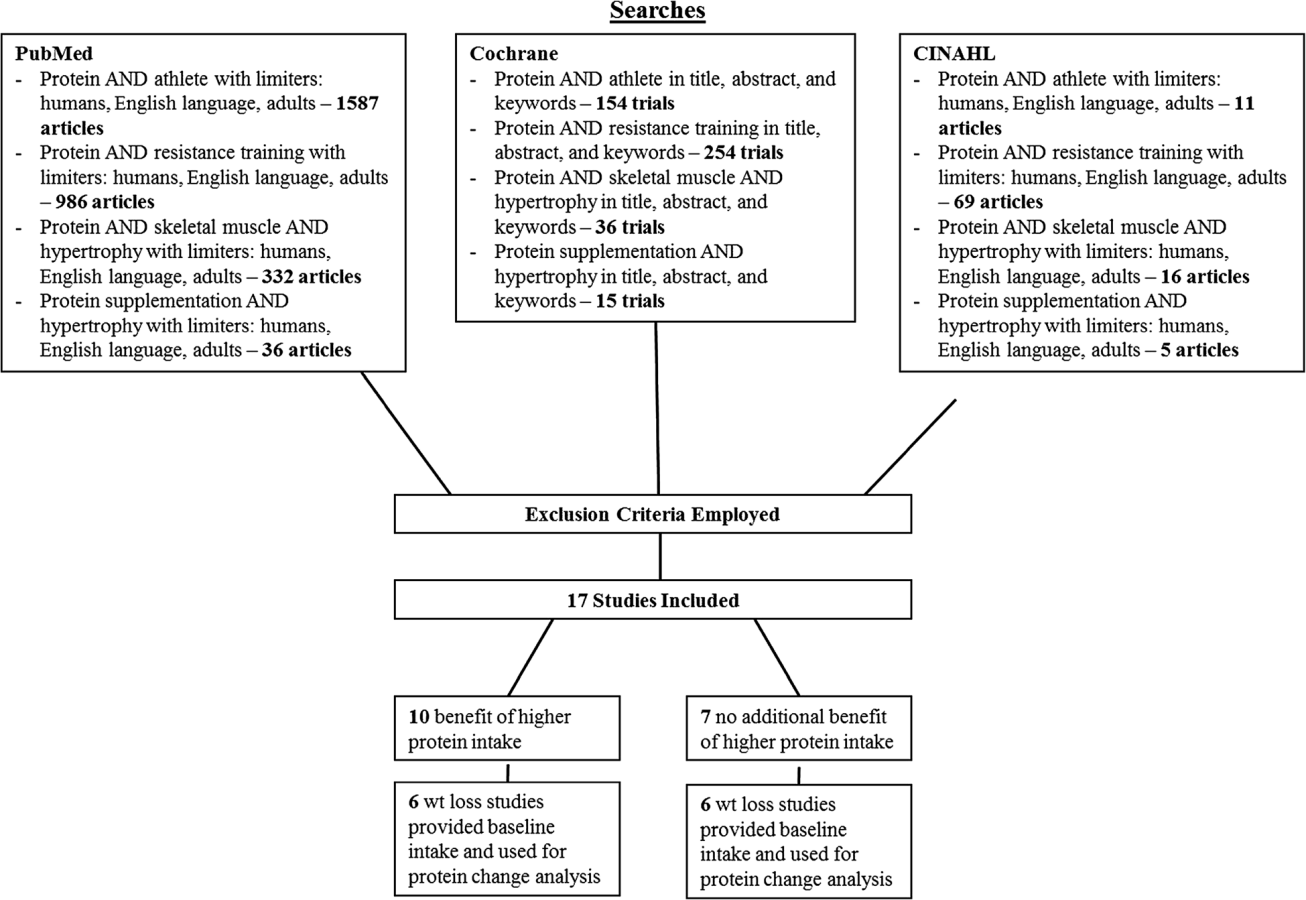
**Division of studies on “protein spread” and “protein change” theories and resistance training.** 1 Reason for exclusion listed only once – some studies may have been excluded for meeting multiple exclusion criteria.

Keyword searches in the PubMed, Cochrane Central Register of Controlled Trials, and CINAHL databases were conducted up to August 2012 using the search criteria in Figure 1. Along with the database searches, reference lists of four major reviews relating to the subject matter were scanned for additional studies to include [11-14]. Before and after exercise have been identified as important times for mediating the effects of nutrition on resistance training gains [15,16]. Some studies in this review provided protein supplements at these times such that control participants did not receive protein at one or both of these times. However, studies with this timing/amount design still typically had a large spread and increase in total daily protein intake from habitual intake. Studies in which 1) spread and change in habitual intake were not manipulated; 2) total protein intake was held constant; and 3) timing was the sole focus were excluded. The decision was made to include timing studies that did manipulate total protein intake since they were present in both groupings of studies where additional protein was and was not more beneficial than control [10,17-20]. Additionally, since data show an elevated muscle protein synthetic response for > 24 hours after resistance training [21], prompt timing of post-exercise protein is likely only one of several predictors of muscle protein accrual following resistance exercise. 

In summary, the following were reasons for exclusion from this review: 1) poor dietary control or reporting; 2) duration < 4 wk; 3) protein timing or type was the primary variable while total intake was held constant; 4) significant differences in baseline characteristics; 5) only one side of the body resistance trained. Based upon the aforementioned criteria, 17 studies were included and reviewed (Table 1).

**Table 1:** Summary of 17 studies reviewed on protein and resistance training

	**Baseline**				**During study**						**Change**											
**Reference**	**BW**	**% BF**	**Protein**	**E**	**Sex**	**Wk**	**Protein**	**Protein**	**E**	**TrS**	**FFM**	**LM**	**% BF**	**Fat mass**	**BW**							
	***kg***	***%***	***g/kg***	***kcal***			***g/kg***	***type***	***kcal***		***kg***	***kg or %***	***%***	***kg***	***kg***							
Burke, 2001 [1]	NR	NR	NR	NR	M	6	1.2	Mix	3240	Tr	NR	0.9	NR	−0.2	1
	NR	NR	NR	NR	M	6	3.3	↑W	3669	Tr	NR	2.3	NR	−0.6	1.5
	NR	NR	NR	NR	M	6	2.2	↑W,Cr	3269	Tr	NR	4	NR	−0.4	3.7
Candow, 2006 [2]^3^	69.3 ± 12	NR	NR	NR	M,F	6	1.7	Mix	3403	UT	NR	0.3	NR	NR	NR
	71.8 ± 15	NR	NR	NR	M,F	6	3	↑S	3415	UT	NR	1.7	NR	NR	NR
	69.3 ± 12	NR	NR	NR	M,F	6	2.95	↑W	3403	UT	NR	2.5	NR	NR	NR
Candow, 2006 [23]^1-3^	87.2 ± 5.8	NR	NR	NR	M	12	1.38	Mix	2878	UT	NR	1 ± 1.3	NR	NR	NR
	87.5 ± 6.4	NR	NR	NR	M	12	1.52	↑LactOv	2630	UT	NR	1.7 ± 1	NR	NR	NR
	85.3 ± 3.6	NR	NR	NR	M	12	1.39	↑LactOv	2753	UT	NR	1.2 ± 0.7	NR	NR	NR
Consolazio, 1975 [3]	NR	NR	1.44	3084	M	6	1.39	C	3452	NR	NR	1.21	NR	−1.09	NR
	NR	NR	1.44	3084	M	6	2.76	C	3532	NR	NR	3.28	NR	−2.21	NR
Cribb, 2007 [4]^1,3^	76 ± 12	16.9 ± 2.4	1.6	2782	M	12	1.65	Mix	2869	Tr	NR	0.7	0.7	0.8	1.4
	70 ± 11	14.9 ± 1.7	1.6	2900	M	12	3.15	↑W	2879	Tr	NR	2.3	0.1	0.4	2.6
	84 ± 14	19.1 ± 1.9	1.5	3536	M	12	3	↑Cr	3313	Tr	NR	4.3	−0.3	0.4	4
	84 ± 12	18.5 ± 1.9	2.1	3423	M	12	3.3	↑W,Cr	3473	Tr	NR	3.4	0	0.7	4
Demling, 2000 [5]^1,3^	NR	27 ± 1.8	0.76	2350	M	12	0.83	Mix	2167	Tr	NR	−0.4 ± 0.4	−2	−2.5 ± 0.5	−2.5 ± 0.6
	NR	26 ± 1.7	0.71	2300	M	12	1.41	↑C	2167	Tr	NR	−4.1 ± 1.4	−8	−7 ± 2.1	−2.8 ± 0.6
	NR	27 ± 1.6	0.73	2350	M	12	1.44	↑W	2183	Tr	NR	−2 ± 0.7	−4	−4.2 ± 9	−2.3 ± 0.5
Eliot, 2008 [22]^2,4^	98 ± 7.6	27.9 ± 1.7	0.94	2175	M	14	0.96	Mix	2188	NR	−0.4	NR	−0.3	−0.6	0.3
	91.1 ± 5.2	28.7 ± 1.4	0.92	1950	M	14	0.84	↑Cr	2012	NR	2.5	NR	−1.2	−0.3	1.3
	88.3 ± 4.4	24.5 ± 1.8	0.95	2010	M	14	0.97	↑W	1938	NR	0.7	NR	−0.3	0	0.4
	92.6 ± 5.1	25.1 ± 1.5	1.03	2007	M	14	1.18	↑W,Cr	2130	NR	1.6	NR	−0.3	0	−0.1
Hartman, 2007 [6]^1,2^	80.5 ± 3.8	NR	1.4	3033	M	12	1.65	Mix	3273	UT	2.4	NR	NR	−0.5	1.9
	83.3 ± 4.1	NR	1.2	3105	M	12	1.65	↑S	2974	UT	2.8	NR	NR	−0.2	2.6
	78.8 ± 2.5	NR	1.4	3009	M	12	1.8	↑Milk	3189	UT	3.9	NR	NR	−0.8	3.1
Hoffman, 2007 [7]^2,3^	99 ± 10.2	21.8 ± 7.3	NR	NR	M	12	1.24	Mix	3139	Tr	NR	0.1 ± 1.4	0.2 ± 1.5	NR	0.4 ± 2
	94.7 ± 7.9	21.7 ± 5.5	NR	NR	M	12	2	↑LactOv	3072	Tr	NR	1.4 ± 1.9	−0.8 ± 2	NR	0.9 ± 1.8
Hulmi, 2009 [8]^1-3^	74.8 ± 8.4	16.6 ± 4.4	1.3	2293	M	21	1.5	Mix	2544	UT	NR	NR	NR	NR	NR
	76.5 ± 7.3	17.1 ± 3.8	1.4	2484	M	21	1.71	↑W	2472	UT	NR	NR	NR	NR	NR
Kerksick, 2006 [9]^1^	85.1 ± 11	17.5 ± 6.1	1.6	3387	M	10	1.56	Mix	2883	Tr	0	0	0	0.2	0.2
	85.3 ± 14.8	18.8 ± 7.3	2.3	3310	M	10	2.12	↑W,AA	2970	Tr	−0.1	−0.1	0.2	0.2	0
	81.2 ± 12.7	17.3 ± 6.4	2.1	2501	M	10	2.32	↑W,C	2736	Tr	1.8	1.9	−0.2	0.1	3
Kukuljan, 2009 [20]^1^	85.2 ± 10.9	28.3 ± 5.5	1.32	2361	M	78	1.31	Mix	2468	UT	NR	0.3	NR	−0.5	0
	83.2 ± 11.9	28 ± 7.8	1.26	2315	M	78	1.4	↑Milk	2400	UT	NR	1.2	NR	−0.6	0.6
Mielke, 2009 [25]	72.4 ± 11.5	19.2 ± 8.5	1.29	2495	M	8	1.15	Mix	2156	UT	−0.3	NR	0.7	0.5	0.1
	79.6 ± 18.1	20.6 ± 7.3	1.36	2632	M	8	1.31	↑W,AA	1988	UT	0.3	NR	0.8	0.4	0.6
Rankin, 2004 [19]	79.8 ± 4.9	20.3 ± 1.5	1.3	2909	M	10	1.2	Mix	2575	UT	0.8	NR	−1.4	−1.3	−0.9
	78 ± 5.2	17.9 ± 2.1	1.2	2488	M	10	1.3	↑Milk	2683	UT	1.6	NR	−0.9	−0.6	0.9
Verdijk, 2009 [18]	80.2 ± 3.4	23.6 ± 2.2	1.1	2197	M	12	1.1	Mix	2173	UT	NR	0.6	−0.7	NR	−0.1
	79.2 ± 2.8	24.9 ± 1.4	1.1	2221	M	12	1.1	↑C	2245	UT	NR	0.7	−1.2	NR	−0.3
White, 2009 [24]^4^	63.6 ± 6.3	31 ± 6	0.88	1603	F	8	0.87	Mix	1466	UT	1.9	NR	−1.4	−0.9	0
	61.7 ± 7.3	29.6 ± 6.2	0.89	1612	F	8	0.96	Mix	1494	UT	1.5	NR	−0.9	−0.2	1.1
	70.8 ± 11	32.8 ± 7.2	0.89	1546	F	8	1.09	↑Milk	1813	UT	2	NR	−1.8	−0.9	1.1
Willoughby, 2007 [10]^1,3^	78.63 ± 13.64	19.95 ± 6.94	2.06	2897	M	10	2.21	Mix	3203	UT	2.7 ± 1.31	NR	−1.07 ± 1.16	−0.22 ± 0.24	4.35 ± 2.88
	81.46 ± 15.78	21.52 ± 7.14	2.21	3569	M	10	2.57	↑W,C	3658	UT	5.62 ± 0.98	NR	−2.06 ± 0.39	−1.13 ± 0.82	7 ± 2.32

^1^ Intake data reported for multiple time points were averaged.

^2^ Denotes study providing additional protein/energy on only resistance training days – additional protein/energy dose divided over 7 days and this was added to the daily average.

^3^ Significant benefit of additional protein to strength and/or muscle CSA/myofibrilar protein.

^4^ Multiple LP and HP groups; data for each protein level were averaged since significant differences were observed or not observed between all LP and HP levels

AA, amino acids; C, casein; Cr, creatine; E, energy; HP, higher protein group; LactOv, milk and egg protein supplement; LP, lower protein or control group; Milk, increased milk consumption; Mix, mixed diet with varied protein sources; NR, not reported; S, soy; Tr, resistance trained participants; TrS, training status; UT, untrained participants; W, whey; Wk, weeks.

Some studies provided protein intake data in g/kg/day terms. When only % energy from protein was provided, the following calculations were made to convert this value into g/kg/day:

1) $\text{g protein}=\left[\left(\left(\%\;\text{energy from protein}\times 0.01\right)\times \text{energy intake}\right)/4\text{kcal}/\text{g}\right]$

2) g/kg/day protein=g protein/kg participant at baseline

When only g protein/day was provided, baseline body mass was the divisor, yielding g/kg/day. When the three macronutrient intakes were provided in g/kg/day format, without energy intake provided, energy intake was obtained by multiplying g/kg/day fat by 9 kcal/g and g/kg/day protein and carbohydrate by 4 kcal/g. This resulted in a kcal/kg/day figure which was multiplied by baseline body mass to obtain total energy intake. When energy intake was provided in mega joules or kilojoules, these numbers were converted and rounded to the nearest kcal. Original dietary intake data sets for multiple time points during studies were often combined as a composite as deemed appropriate and are noted (Table 1). Most studies provided daily supplementation of protein, however, for studies providing supplemental protein on resistance training days only, the total supplemental protein consumed per week was divided by seven days and added to the mean reported daily intakes. The protein intakes provided in this review include all food and supplementation consumed.

The term “higher protein” was used in this review to describe the group within a study that had a “higher protein” intake relative to a “lower protein” group, sometimes referred to as a “control” group. “Higher” and “lower” were relative, not denoting a specific level of intake. Additionally, original intake data sets for multiple time points during studies were often combined as a composite when deemed appropriate (Table 1). Finally, studies which showed benefits from two types of protein supplementation had the protein intake levels of these two groups averaged as the “higher protein” group for spread calculations. “Spread” calculations for protein spread theory were calculated by:

(1) $$\begin{array}{l} \text{Between group}\;\%\;\text{spread in protein intake}=[((\text{higher protein group g}/\text{kg}/\text{day intake}\;\\ \text{during study}-\text{control group g}/\text{kg}/\text{day intake during study})/\text{control group g}/\text{kg}/\text{day intake during study})\times 100] \end{array}$$

“Change in habitual protein intake” calculations were calculated by:

(2) $$\begin{array}{l} \text{Change in habitual protein intake}=[((\text{g}/\text{kg}/\text{day intake}\;\\ \text{during study}–\text{g}/\text{kg}/\text{day intake at baseline})/\text{g}/\text{kg}/\text{day intake at baseline})\times 100] \end{array}$$

For both theories, after these values were obtained for each study, means of these values for groups of studies were calculated for analysis. Clarification on dietary intake data was obtained by contacting authors [6,8,9] as necessary.

## Results

Ten of the 17 studies [1-10] showed superior muscular benefits of a higher protein intake over control (Figure 1). However, seven studies [18-20,22-25] meeting inclusion criteria showed no greater muscular benefits of a higher protein intake compared to control. Thus, we proposed protein spread and change theory as possible explanations for this discrepancy.

### Protein spread theory

**Figure 2:**
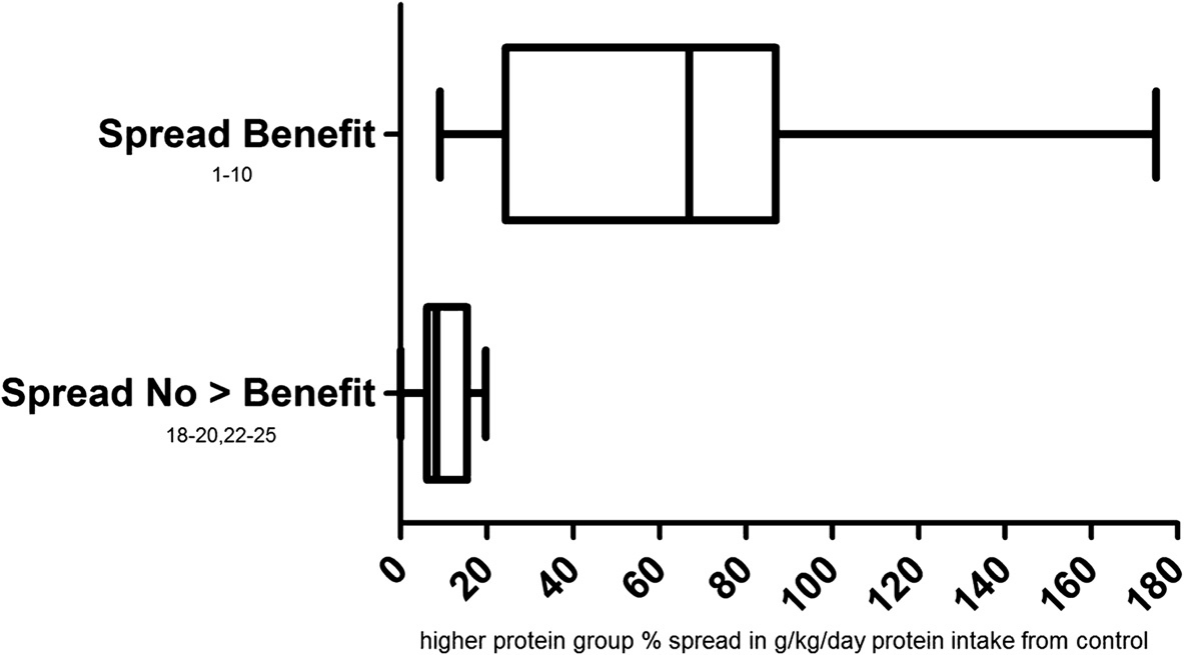
**Spreads in protein consumption between higher and lower protein groups in protein spread analysis.** Spread Benefit = those studies in which the higher protein group experienced greater muscular benefits than controls during the intervention; Spread No > Benefit = those studies in which the higher protein group experienced no greater muscular benefits than controls during the intervention.

Within ten studies showing muscular benefits of a higher protein intake (Figure 2), g/kg/day protein intake was 66.1% greater than control on average (Table 2). For example, Hoffman et al. had resistance trained football players consume either 2 or 1.24 g/kg/day protein during 12 wk resistance training. Maximum squat strength increases were significantly greater (23.5 kg) in the higher protein group versus controls (9.1 kg) [7]. Cribb et al. had resistance trained men consume 3.15 g/kg/day or 1.65 g/kg/day protein during an 11 wk resistance training program. The higher intake was achieved via whey protein isolate supplementation and this group gained significantly greater strength and myofibrillar protein in the quadriceps than control [4]. Whey and soy protein supplementation was also used by Candow et al. to bring two groups of participants to a daily intake of ~3 g/kg/day versus 1.7 g/kg/day in controls. After six wk resistance training, the lean mass gains of 2.5 and 1.7 kg in the whey and soy groups were significantly greater than the 0.3 kg gain in controls. Squat and bench press strength increased ~25 and 8 kg respectively in the higher protein groups which was significantly greater than the control gains of ~14 and 4 kg [2]. Similarly, resistance trained participants in a study by Burke et al. achieved a 3.3 g/kg/day protein intake via whey protein supplementation compared to 1.2 g/kg/day in controls. During six wk of resistance training this led to a 2.3 kg gain in lean body mass along with a 16.5 Nm gain in isokinetic knee extension peak torque. Both results were statistically significant while the gains of 0.9 kg and 11.6 Nm of the same measures in the control group were not significant [1]. On the other hand, the mean g/kg/day protein intake in the higher protein groups in six studies showing no additional muscular benefits of higher protein (Figure 2) was only 10.2% greater than controls on average. 

**Table 2:** Percent spread in protein intake between groups in studies included in protein spread theory analysis

**Benefit**		**No > benefit than control**	
**Study**	**% Spread (g/kg/day)**	**Study**	**% Spread (g/kg/day)**
Burke, 2004 [1]	175	Candow, 2006 [23]	5.8
Candow, 2006 [2]	75	Eliot, 2008 [22]	19.7
Consolazio, 1975 [3]	98.6	Kukuljan, 2009 [20]	6.5
Cribb, 2007 [4]	90.9	Mielke, 2009 [25]	13.8
Demling, 2000 [5]	72.6	Rankin, 2004 [19]	8.3
Hartman, 2007 [6]	9.1	Verdijk, 2009 [18]	0
Hoffman, 2007 [7]	61.3	White, 2009 [24]	17.1
Hulmi, 2009 [8]	14		
Kerksick, 2006 [9]	48.7		
Willoughby, 2011 [10]	16.3		
Average % Spread (g/kg):	66.1	Average % Spread (g/kg):	10.2

### Protein change theory

**Figure 3:**
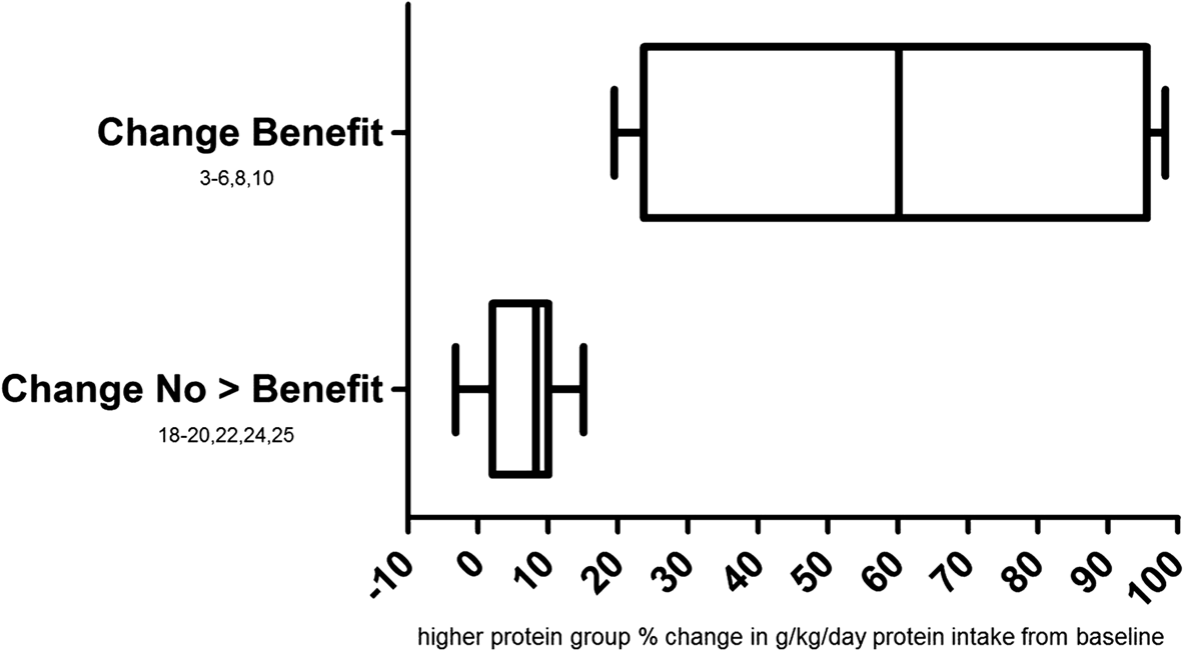
**Percent deviation from habitual protein intake among groups in protein change analysis.** Change Benefit = those baseline reporting studies in which the higher protein group experienced greater muscular benefits than controls during the intervention; Spread No > Benefit = those baseline reporting studies in which the higher protein group experienced no greater muscular benefits than controls during the intervention.

Not all studies reported baseline dietary intake. Of the twelve that did (Figure 3), the average percent increase in habitual g/kg/day protein intake was 6.5% in six studies that showed no additional benefit compared to 59.5% in six studies which showed muscular benefits to a higher protein intake (Tables 3 and 4). In the protein change analysis, all studies that showed muscular benefits of increased protein intake involved an increase in habitual protein intake of at least 19.5%. As two of six examples, the studies by Cribb et al. and Demling et al. which also supported protein spread theory involved changes in habitual protein intake of 97-98% [4,5]. This led to greater muscular benefits in both studies. The six studies that showed no additional muscular benefits from protein supplementation also followed the postulations of our theories. For example, untrained participants of a study by Rankin et al. consumed either 1.3 g/kg/day protein or 1.2 g/kg/day protein. The 1.3 g/kg/day group followed an intervention of increased milk intake, yet only increased their habitual protein intake by 8.33%. Ten weeks of resistance training led to similar strength and body composition improvements in both groups [19]. Similarly, there were no muscle or strength differences between participants consuming 1.31 g/kg/day protein via additional milk compared to non-milk supplementing participants consuming 1.28 g/kg/day protein daily in a study by Kukuljan et al. [20]. 

**Table 3:** Protein change theory studies showing muscular benefits of increased protein versus control

** Study**	**LP base intake (g/kg/day)**	**LP study intake (g/kg/day)**	**HP base intake (g/kg/day)**	**HP study intake (g/kg/day)**	**LP change (%)**	**HP change (%)**
Consolazio, 1975 [3]	1.44	1.39	1.44	2.76	−3.5	91.7
Cribb, 2007 [4]	1.6	1.65	1.6	3.15	3.1	96.9
Demling, 2011 [5]	0.76	0.83	0.72	1.43	9.5	98.2
Hartman, 2007 [6]	1.4	1.65	1.4	1.8	17.9	28.6
Hulmi, 2009 [8]	1.3	1.5	1.4	1.71	15.4	22.1
Willoughby, 2007 [10]	2.06	2.21	2.15	2.57	7.3	19.5
Average % Change (g/kg):					8.3	59.5

HP, higher protein; LP, lower protein.

**Table 4:** Protein change theory studies showing no > muscular benefits of increased protein versus control

** Study**	**LP base intake (g/kg/day)**	**LP study intake (g/kg/day)**	**HP base intake (g/kg/day)**	**HP study intake (g/kg/day)**	**LP change (%)**	**HP change (%)**
Eliot, 2008 [22]	0.93	0.9	0.99	1.07	−3.3	8.3
Kukuljan, 2009 [20]	1.32	1.31	1.26	1.4	−0.8	10.7
Mielke, 2009 [25]	1.29	1.15	1.36	1.06	−10.6	−3.2
Rankin, 2004 [19]	1.3	1.2	1.2	1.3	−7.7	8.3
Verdijk, 2009 [18]	1.1	1.1	1.1	1.1	0	0
White, 2009 [24]	0.88	0.87	0.89	1.02	−0.9	15.1
Average % Change (g/kg):					−3.9	6.5

HP, higher protein; LP, lower protein.

## Discussion

This review supports our protein spread and change theories [11] as possible explanations for discrepancies in the protein and resistance training literature. In our previous review, we demonstrated that spread and change in study protein intakes may be important factors predicting potential to benefit from increased protein during a weight management intervention. In studies from the present review that showed greater muscular benefits of higher protein, there was a greater % spread between the g/kg/day intake of the higher protein group and control. Additionally, that the higher protein group’s during study g/kg/day protein intake is substantially different than baseline is important. With minimal spreads and changes from habitual intake there are little additional muscular benefits from higher protein interventions. Evidence weighs heavily toward muscular benefits from increased protein [1-10]. Those studies that did not support additional benefits of greater protein still showed that higher protein was as good as an alternative diet [18-20,22-25].

### Protein spread theory

Protein type influences the acute anabolic response to resistance training [26] and cannot be overlooked as a possible influence on protein spread theory results. Trained participants in a 10 wk study by Kerksick et al. reached ~2.2 g/kg/day protein from whey/casein protein or whey/amino acid supplementation. Controls consumed 1.56 g/kg/day. Only the whey/casein group gained significantly greater (1.9 kg) lean mass than controls [9]. Hartman et al. had untrained participants supplement with soy protein or milk to achieve a protein intake of 1.65 and 1.8 g/kg/day. Controls consumed 1.65 g/kg/day. The milk group achieved significantly greater increases in type II and I muscle fiber cross-sectional area than controls; soy gains were only significantly greater than controls for type I [6]. These results [6,9] make more sense in the context of protein spread theory. That is, Kerksick et al.’s whey/casein group achieved a 12.8% g/kg/day greater spread from controls than did the whey/amino group [9]. Hartman et al.’s milk group achieved a 9.1% g/kg/day spread versus controls; the soy group consumed the same as controls [6]. Protein type, whey or soy, did not affect lean mass and strength gains in a study by Candow et al. [2] where there was no spread in protein intake between supplementation groups.

Similar to the Kerksick et al. study, lean mass gains, strength gains, and fat loss in participants supplementing with casein protein from Demling et al. were significantly greater than in the whey protein group [5], however the spreads and changes were essentially identical for the casein and whey groups [5]. These authors suggested that perhaps the slow digestion of the casein protein enhanced nitrogen retention as shown previously [27] and this nitrogen retention led to greater muscular gains over time. This explanation was also presented by Kerksick et al. [9]. The influence of acute post-exercise protein kinetics on long-term gains obtained from supplementation during resistance training warrants further research.

None of the “no greater benefits” studies were outside of normal distribution. However, three studies [22,24,25] had spreads that were higher than three studies [6,8,10] of the “muscular benefits” grouping. These seemed likely explained, however, by the fact that changes to habitual protein intake were much larger in the latter [6,8,10] than the former [22,24,25].

#### Protein change theory

Only twelve studies included in this review reported baseline dietary intakes. Among studies showing muscular benefits of increased protein intake, the three with the smallest increases from habitual protein intake (19.5-28.6%) were conducted on untrained participants [6,8,10]. Most studies were on trained participants and larger increases in protein intake. However the ~4 kcal/kg greater energy intake in one of these studies [10] or perhaps the longer duration of another study [8] may have made it easier for a smaller change to yield significant results. That said, total energy intake was higher in some higher protein groups than control and lower than control in other studies (Table 1) making it hard to use energy intake as a clear predictor of results.

Further supporting higher habitual protein intake during resistance training, Ratamess et al.’s strength/power athletes consuming 2.3 g/kg/day were significantly leaner than those consuming 1.45 or 0.95 g/kg/day [28]. While monitored for 10 wk, the 2.3 g/kg/day group consumed ~400-700 kcal or ~6-10.5 kcal/kg/day more than the other tertiles, yet remained significantly leaner by ~5-8% bodyfat. Strong correlations have been shown between increased habitual protein intake [29], regular ingestion of quality protein [30], and muscle mass. In contrast, Thalacker-Mercer et al., found no association between habitual protein intakes of 0.97-1.07 g/kg/day and muscular gains [31]. However, since Ratamess et al. showed no differences between 0.95 and 1.45 g/kg/day [28], it seems unlikely that 0.97 versus 1.07 g/kg/day was enough difference to see a protein effect [31]. Variability in resistance training volume (1–5 sets/exercise), intensity (3–20 RM), and frequency (3-5- day/wk) across studies in this review may also have interacted with response to protein supplementation. However, most studies used resistance training variables in the middle of these ranges and there was no pattern of a greater frequency of training programs employing certain variables within the benefits or no greater benefits groupings. Since protein benefits muscle mass in lieu of resistance training [32,33], even if a training program was suboptimal, a higher protein intake should still offer a statistically significant benefit over a lower intake.

The findings of Ratamess et al. and Thalacker-Mercer et al. [28,31] bring scientific backing to a common phenomenon: that nutritional and training recommendations based upon group means are not effective for all individuals. Data displayed by Lockwood et al. on a per participant basis demonstrates this [34]. Determining the genetic, epigenetic, and other factors influencing variability in response to nutrition/training is the future of sports nutrition.

Age may impair the acute anabolic response to protein with resistance exercise [35], although this finding is not universal [36] and could also be complicated by protein type. Although minimal change or spread in protein intake was achieved in groups of two studies not showing a benefit of greater protein [18,20], perhaps age was a factor in this lack of response. However, this would seem to point more convincingly toward protein change theory; perhaps creating a more pronounced change from habitual intake in older populations is even more important than in younger populations. New related data support this [37].

### Application of this review in resistance training

If a nutrition professional met with two clients with near identical anthropometrics, one consuming 0.97 g/kg/day protein versus another consuming a strength/power athlete recommended level of 1.45 g/kg/day, the practitioner might assume given equal energy intake, that the athlete consuming 1.45 g/kg/day had an anabolic advantage. While a valid generalization, Ratamess et al.’s data do not support it [28]. If amidst other factors promoting anabolism this 1.45 g/kg/day client was not gaining lean mass, surely the practitioner would not tell them his/her cause was hopeless. However, recommending an increased dietary protein would be deemed of little benefit by many nutrition professionals, yet data continually show contrary [1-7,9,10,17,28,38].

Often studies examining protein type or timing are viewed solely for these variables and do not address spread in total intake or change from habitual intake. In several studies, controls consumed protein at ~1.5-2.5 times the current RDA, in line with current strength/power recommendations, yet in many cases, adding additional protein produced significantly greater muscular benefits [1,2,4,6,9]. That protein at current recommendations for strength/power was less beneficial that even more protein is perhaps explained as: 1) protein recommendations are largely based on nitrogen balance studies, which fail to address a level of protein to optimize body composition [39]; 2) per protein habituation theory, increasing a typical American intake of ~1 g/kg/day [40,41], to strength/power athlete recommendations of 1.4-1.8 g/kg/day provides sufficient deviation from habitual intake. Meanwhile, resistance training participants from this review were shown to consume 1.31 g/kg protein habitually. Thus, achieving this same deviation of 40-80% from habitual protein intake would dictate protein intakes of 1.83-2.36 g/kg, which are greater than current strength/power recommendations.

The body’s response to protein is not static, but adjusts to the diet it is afforded [42-44]. For example, progressive increases in protein intake are coupled with increased fasting nitrogen losses [45,46] along with an increase in feeding induced nitrogen accrual [45,46] that is perhaps even more pronounced than fasting losses [45]. Although not fully elucidated, a possible implication of this might be an effect on lean tissue mass. A few studies specifically address change in habitual protein intake. Soenen et al. had participants increase habitual protein intake 16%, from 1.13 g/kg/day to 1.31 g/kg/day via substitution of ~500 kcal with a milk protein based supplement containing 52 g protein. Over 12 weight-stable wk this led to 0.7 kg greater lean mass gain and fat loss compared to isoenergetic controls [33]. Bray et al. reported that increasing a 1.2 g/kg/day protein intake to ≥ 1.8 g/kg/day via overfeeding led to an ~3.5-4 kg greater gain in lean body mass in eight wk [32]. Additionally, Petzke et al. reported a positive correlation (r = 0.643, p = 0.0001) between change in habitual protein intake and change in fat-free body mass [29]. Habitual intake mediates the effects of protein on bone health and satiety [47,48] and studies have shown that that the thermic effect of protein decreases over time while dieting [49,50]. We propose that changes in habitual protein intake may mediate the effects of protein on lean body mass [29]. Finally, it is likely that adding protein to one’s habitual intake is most beneficial when added to previously protein poor meals, as opposed to adding to meals already highin protein [51,52]. Protein distribution should also be accounted for in future research.

## Conclusions

Baseline protein intakes averaged ~1.31 g/kg/day (Tables 3 and 4), short of the mean high protein group intake during studies showing muscular benefits of 2.38 g/kg/day. Per protein change theory, a 59.5% increase to a representative habitual protein intake of ~1.31 g/kg/day would yield 2.09 g/kg/day. This is close to the aforementioned 2.38 g/kg/day benchmark. The “lay” recommendation to consume 1 g protein/lb of bodyweight/day (2.2 g/kg/day) while resistance training has pervaded for years. Nutrition professionals often deem this lay recommendation excessive and not supported by research. However, as this review shows, this “lay” recommendation aligns well with research that assesses applied outcome measures of strength and body composition in studies of duration > 4 weeks [1-7,9,10,17,28,38]. That current sports nutrition guidelines for resistance training continue to mirror results of nitrogen balance studies [53,54], is perhaps not optimal.

Higher protein interventions were deemed successful when there was, on average, a 66.1% g/kg/day between group intake spread compared to 10.2% when additional protein was no more effective than control. The average change in habitual protein intake in studies showing higher protein to be more effective than control was +59.5% versus +6.5% when additional protein was no more effective than control. These findings support our protein spread and change theories in a sports nutrition context. In the same respective order, the four means from our weight management review on these theories were 58.4%, 38.8%, 28.6%, and 4.9% [11].Thresholds or specific numbers for application of these theories are likely context specific. However, the general magnitude differences between studies showing muscular benefits and no benefits of additional protein appear repeatable across studies and aid in moving toward individualized protein recommendations. Consideration of these theories is encouraged in the design of future trials.

## Abbreviations

RDA: Recommended dietary allowance; g/kg/day: Grams protein per kilogram per day.

## Competing interests

JDB and BMD are employees of USANA Health Sciences, Inc. USANA Health Sciences, Inc. had no role in the direction, data collection, analysis, interpretation, or writing of this review. USANA Health Sciences, Inc. has provided for the article processing charge. The authors have no other competing interests to declare.

## Authors’ contributions

JDB designed the manuscript, collected and analyzed study data, wrote, and edited the manuscript. BMD provided manuscript direction and edited the manuscript. Both authors read and approved the final manuscript.

## Authors’ information

JDB holds an MS in Sports Dietetics, a BS in Exercise Science and is a Registered Dietitian and Senior Scientist for USANA Health Sciences, Inc. JDB is an Adjunct Professor to graduate students in the Division of Nutrition at the University of Utah. JDB has worked in the field with weight management clientele, collegiate, and professional athletes and in the lab researching shoulder biomechanics and the role of macronutrients in hypertension. Having reviewed protein metabolism literature, JDB’s current objective is to provide insight on scientific research based upon phenomena observed by practitioners in the field. BMD holds a PhD in Molecular and Cellular Biology from Oregon State University and has published numerous original scientific studies, most recently on the role of vitamin D in active populations. As Executive Director of Product & Technology Innovation, BMD oversees an expansive clinical studies program involving collaborations between USANA Health Sciences and several universities and private research institutions.
